# Trauma informed weight lifting: considerations for coaches, trainers and gym environments

**DOI:** 10.3389/fpsyg.2023.1224594

**Published:** 2023-07-20

**Authors:** Dana Vigue, Mariah Rooney, Eva Nowakowski-Sims, Savannah Woods

**Affiliations:** ^1^Department of Anthropology, Harvard Graduate School of Arts and Sciences, Harvard University, Cambridge, MA, United States; ^2^Harvard Medical School, Boston, MA, United States; ^3^Center for Trauma and Embodiment, Justice Resource Institute, Needham, MA, United States; ^4^School of Social Work, Barry University, Miami Shores, FL, United States; ^5^The New School for Social Research, New York, NY, United States

**Keywords:** weight lifting, trauma, mind-body connection, qualitative research, mental health, exercise

## Abstract

A growing body of research supports weight lifting as an effective adjunct intervention in the treatment of psychological trauma and trauma-related disorders. However, studies indicate that numerous barriers exist to participation in weight lifting, especially among populations disproportionately impacted by trauma. Trauma-informed care offers a paradigm for service delivery that aims to empower clients to access healing resources by attending to six domains of experience: safety, trustworthiness and transparency, peer support, collaboration and mutuality, empowerment, voice and choice, and cultural, historical, and gender issues. This mixed-methods study utilizes semi-structured, one-on-one interviews and surveys to inform the design of an evidence-based trauma-informed weight lifting program for trauma survivors. The contributions of this paper are twofold. First, the salient experiential categories for conceptualizing psychological healing in the gym environment are presented, informed by the lived experiences and recommendations of a national sample of trauma-impacted weight lifters. Second, based on the findings of this study, guidelines are proposed for the development of trauma-informed weight lifting programming that may be incorporated into the practice habits of personal trainers. The results of this study aid in the reduction in barriers that currently limit the adoption of weight lifting as an adjunct intervention for trauma and contribute to the professionalization of personal trainers in trauma-related competencies.

## Introduction

A growing body of research has supported weight lifting as an effective adjunct intervention in the treatment of psychological trauma and trauma-related disorders. Weight lifting has been associated with improvements in symptoms of PTSD (Whitworth et al., 2017, 2019), reductions in substance use (Unhjem et al., 2016), improved self-efficacy (O'Connor et al., 2010), increased social connection, and the formation of empowering self-identity in the aftermath of trauma (Nowakowski-Sims et al., 2023). In addition, weight lifting holds potential to address many persistent barriers to accessing trauma healing resources including mental health resources such as therapy. A recent survey of 2,053 U.S. adults revealed that 42% of respondents who needed mental health care in the past 12 months did not receive it (National Council for Mental Wellbeing, 2022). Over one third of those who went without mental health care cited barriers related to cost, over one quarter could not access mental health care because they could not locate a provider close by, and one quarter could not access care because they could not find a provider who offered a visit format (i.e., in-person, telehealth) with which they were comfortable (National Council for Mental Wellbeing, 2022).

Weight lifting holds promise in addressing these important access issues. Weight lifting can be practiced in a variety of settings and does not require health insurance or a clinician’s referral. Many people in the U.S. are familiar with or already engage in weight lifting. One recent secondary analysis of 99,713 participants recruited for the Prostate, Lung, Colorectal, and Ovarian Cancer Screening Trial demonstrated that nearly 1 in 4 respondents reported some weight lifting activity (Gorzelitz et al., 2022). Over 64 million people in the U.S. have gym memberships (Gough, 2022) with roughly 40% of members paying less than $25 per month for their memberships (IHRSA, 2021). Weight lifting also has broad appeal, as a variety of modalities exist, ranging from the organized sports such as Olympic weightlifting and powerlifting to recreational approaches including circuit training and group strength and interval training classes. In addition, youth and adolescent participation in weight lifting, through weight lifting sports and the use of weight lifting as part of conditioning for other sports, has been steadily increasing (Pierce et al., 2022). Thus, if systematically leveraged as a healing intervention, weight lifting may play an important role in expanding access to trauma healing resources.

However, numerous barriers exist to participation in physical activity in general and weight lifting specifically, especially among populations disproportionately impacted by trauma. For instance, although members of LGBTQ+ communities are overrepresented among those impacted by trauma (Kassing et al., 2021), they experience significant barriers to participating in physical activity– including homophobia, transphobia, exclusion, and discrimination– and, as a result, engage in significantly less physical activity than their cisgender and heterosexual counterparts (Herrick et al., 2022). In addition, research has shown that African Americans face disproportionate rates of trauma exposure (Alim et al., 2006) and are significantly less likely to engage in physical activity than white Americans; a disparity that is exacerbated by social and economic factors such as neighborhood poverty (Hawes et al., 2019). A recent grounded theory study exploring weight lifting as an adjunct intervention for trauma (Nowakowski-Sims et al., 2023) found that multiple factors limit access to weight lifting among trauma survivors, including a lack of gender-inclusive environments, stigma against persons of size, lack of coach familiarity with the sequelae of trauma, and cost of participation. These barriers limit the feasibility of weight lifting as an adjunct treatment for trauma, despite evidence of its efficacy. These important access issues must be addressed if weight lifting is to be leveraged as an adjunct healing intervention for trauma.

One promising approach to increase the accessibility of weight lifting for trauma survivors involves taking a trauma-informed approach to weight lifting programming. Trauma-informed approaches to care delivery were first introduced by Harris and Fallot (2001) in response to the high prevalence of sexual and physical abuse among people seeking mental health services. In contrast with trauma-specific services, “to be trauma informed means to understand the role that violence and victimization play in the lives of most consumers of mental health and substance abuse services and to use that understanding to design service systems that accommodate the vulnerabilities of trauma survivors and allow services to be delivered in a way that will facilitate consumer participation in treatment” (p. 4). Today, the Substance Abuse and Mental Health Services Administration (SAMHSA, 2013) endorses the use of six trauma-informed care principles: safety, trustworthiness, and transparency; peer support; collaboration and mutuality; empowerment, voice, and choice; and cultural, historical, and gender issues. Because of the high prevalence of trauma, with 70% of adults in the U.S. reporting experiencing a traumatic event in their lifetimes (How To Manage Trauma, 2013), health care practitioners have proposed that trauma-informed approaches to care be adopted as universal precautions, akin to other standardized protective measures in health care such as hand hygiene (Racine et al., 2020). Universal trauma precautions transform service delivery systems to promote the psychological safety of clients when accessing health-promoting resources. Trauma-informed approaches have since been adopted across a diverse range of sectors, including education, juvenile detention, and workplaces.

More recently, the role of trauma-informed approaches to exercise and sport has been explored. Clark et al. (2014) demonstrated that a trauma-sensitive approach to yoga instruction for survivors of domestic violence was feasible, well-received, and significantly reduced PTSD symptoms about as effectively as current approaches using therapy or medications. Rosenblum and Taska (2014) showed that self-defense training informed by trauma research and delivered in collaboration with trauma therapists served as an effective adjunct to treatment among female survivors of trauma with PTSD, anxiety, and depressive disorders. Recent calls have been made for increased qualitative work to guide the trauma-informed implementation of other exercise modalities (Pebole Gobin and Hall, 2021). However, at present, there are no evidence-based guidelines for a trauma-informed approach to instruction in weight lifting.

This mixed-methods study aims to address the unmet need for evidence-based guidelines for a trauma-informed weight lifting program in order to increase access to weight lifting as an adjunct health resource for psychological trauma and trauma-related disorders. This study explores participant experiences with gym environments and their recommendations for spaces that are trauma-informed. The contributions of this paper are twofold. First, the salient experiential categories for conceptualizing psychological healing in the gym environment are presented, informed by the lived experiences and recommendations of a sample of trauma-impacted weight lifters. Second, based on the findings of this study, guidelines are proposed for the development of trauma-informed weight lifting programming that may be incorporated into the practice habits of personal trainers. The results of this study aid in the reduction in barriers that currently limit the adoption of weight lifting as an adjunct intervention for trauma and contribute to the professionalization of personal trainers in trauma-related competencies.

## Methods

### Participant recruitment

The sample is a non-probability purposive sample that included lifters with a self-identified trauma history. The researchers have a collaborative relationship with The Justice Resource Institute, the parent organization to the Center for Trauma and Embodiment which is home to programs such as Trauma Informed Weight Lifting and Trauma Center Trauma Sensitive Yoga. Following approval from the researchers’ institutional review boards, The Justice Resource Institute sent emails detailing the study and recruitment plan to their email lists and posted a study flier on their social media platforms. Interested participants contacted the researchers to schedule a video interview. The qualifying criteria for participation in the study were a self-reported trauma history and participation in weight lifting for at least 3 months. Institutional review board approval was granted by Barry University and the Justice Resource Institute.

### Data collection

This mixed-methods study utilized both semi-structured, one-on-one interviews and surveys to characterize the experiences of trauma-impacted individuals engaged in weight lifting. The study team consisted of four members, comprised of professors and graduate students in psychology and anthropology who have received formal training in qualitative research methods. All members of the study team engaged in participant recruitment, data collection, and data analysis.

In response to inbound interest via email from prospective research participants, members of the study team introduced themselves as researchers affiliated with the Center for Trauma and Embodiment program, Trauma Informed Weight Lifting, and provided additional information about the study. Research participants completed an informed consent via email prior to participation in the study. The informed consent advised participants as to the purpose of the study, the possible risks of participation, and the capacity and limitations of the video meeting platform Zoom’s privacy features. Semi-structured, one-on-one interviews were carried out over Zoom by members of the study team. Interviews solicited in-depth feedback about the elements of weight lifting participants found to be beneficial, barriers to healing from trauma, and thoughts and feelings about introducing trauma-informed elements in the weight lifting. For a complete list of interview questions, please see Appendix 1. Interviews ranged from 45 to 90 min in length. Each interview was audio-recorded and transcribed verbatim using NVivo software. Each transcript was then reviewed for accuracy by a second member of the study team, and revisions were made if necessary.

In addition to participation in semi-structured interviews, participants were asked to complete an online survey regarding their demographic information and trauma history. Participants self-reported their age, race, gender identity, sexual orientation, level of education, employment status, duration of participation in weight lifting, the type of weight lifting they engaged in (i.e., powerlifting, CrossFit, recreational, free weights), the setting in which they engaged in weight lifting (i.e., home, gym, outside), whether they lifted weights with a coach, the type of coaching they received (i.e., one-on-one, small group, large group), and their city and state of residence. Participants were also asked to indicate the type of trauma they were exposed to. Trauma types included interpersonal trauma occurring in the context of an interaction with another person (i.e., assault), impersonal trauma not involving interaction with another person (i.e., natural disaster), and systemic trauma involving societal or institutional harms (i.e., discrimination). These categories represent an adaptation of Allen’s (2001) framework of interpersonal and impersonal trauma based on growing evidence of the distinct psychological significance of systemic trauma as a third category of experience (Goldsmith et al., 2014). An additional survey field was provided to participants to enter their preferred terminology to describe their trauma history. In addition, participants were asked to indicate whether their trauma exposures were acute (i.e., occurred as a single episode), chronic (i.e., prolonged or ongoing), or both acute and chronic. These categories represent lay adaptations of Terr’s (1991) Type I and Type II trauma. To minimize the risks of psychological distress to participants, specific details about trauma history were not solicited.

### Data analysis

Semi-structured interviews were analyzed using a framework method of analysis (Ritchie and Spencer, 1994) to explore and characterize prominent features of weight lifting described by survivors of trauma. Framework analysis is an inherently comparative form of thematic analysis which employs an organized structure of inductively- and deductively-derived themes to conduct cross-sectional analysis using a combination of data description and abstraction (Ritchie and Spencer, 1994; Goldsmith, 2021).

Researchers ensured study validity with a well-structured data analysis procedure. Open coding was first used to inductively identify salient themes surrounding participant experiences with gym environments. Each researcher independently coded transcripts and met regularly to discuss and resolve differences in interpretations of initial codes. When an analytic discrepancy was identified, the meaning of the narrative was discussed, and only the codes or themes in which there was agreement were selected. In this way, the researchers adhered to a consistent thematic data analysis process and scheme of interpreting collected responses.

Analytic memo writing was used throughout the coding process to make meaning of the initial codes and subsequent themes. Early memos informed the selection of the framework method for the subsequent organization of codes into a theoretical framework. A framework matrix was used to summarize and analyze data in a table of rows and columns, facilitating the identification of salient themes within and across interviews. As an additional measure of credibility, direct and representative quotes from the participants were included to demonstrate each theme.

In early meetings, researchers noticed that the codes found in the data could be organized according to pre-existing theoretical frameworks for trauma-informed care, particularly SAMHSA’s six principles of trauma-informed care: safety; trustworthiness, and transparency; peer support; collaboration and mutuality; empowerment, voice, and choice; and cultural, historical, and gender issues. This theoretical alignment was a pertinent finding of the study, thus the researchers elected to utilize these six analytic categories as an organizing framework during the theorization phase of analysis. Such a choice is congruent with the framework method, in which researchers first use an inductive approach to identify themes in the data, then use a theory deductively to help further explain certain themes. As Gale et al. (2013) explain, such “a combined approach [of induction and deduction] is appropriate when the project has some specific issues to explore, but also aims to leave space to discover other unexpected aspects of the participants’ experience or the way they assign meaning to phenomena” (p. 2). The resultant framework matrix provided a descriptive overview of the entire data set.

Comparative analysis across transcripts within these six analytic categories advanced the analysis from concrete descriptions of themes to the identification of more abstract concepts (Goldsmith, 2021). In this study, researchers moved beyond identifying SAMHSA’s six principles of trauma-informed care within the data set and went on to explicate the ways in which these experiential domains pertained to the experience of weight lifting. Table 1 offers an example of summarized findings within the framework method related to SAMHSA’s principle of safety.

**Table 1:** Framework analysis matrix: safety.

Theme	Sub theme	Example
Not safe	Physical touch	“And also I’ve been in so many weight lifting spaces where coaches or helpful standers by are like touching people’s bodies without their consent. Or spotting in ways that aren’t the only option, but happen to be an option that do not feel safe for that person. And so, you know, having your hands on someone’s waist is by no means the only way to support, you know, spot someone squat. And guess what? I do not want it. So some of the like interpersonal things attending to like what needs someone has in their body.”
Not safe	Physical touch	“people do not always want to be touched and but they do not always want to stand out as the one that’s like waving their hands around saying they do not want to be touched either.”
Not safe	Physical space	“I know for me, I do not like a space where everyone– like a fishbowl– everyone is walking by and looking in. So let us say we have a room that’s designated for a class, but then there’s windows everywhere and everyone’s just walking by and looking in. That is an awful feeling. I do not like that at all. That’s horrible. I do not feel safe in that environment.”
Not safe	Physical space	“the other thing that I do not love is people standing over me while I’m laying down, which can be hard”
Not safe	Physical space	“Really crowded, tight spaces can be hard, I think. But when you do not have your own space to be doing what you are doing, that hypervigilance can be really powerful in those moments. I’m just constantly kind of checking like, am I OK?”
Not safe	Emotional	“And this big old burly man came in to teach a class and he was like a drill sergeant or a Marine boot camp guy. And I just shut down and he kept. Yelling to push and then and there were only three of us in the class, and so he kept saying my name…”
Not safe	Emotional	“I struggled with the idea of the concept of weight lifting when I was younger because it was so cis and het and white, I want to say primarily, and like very cis-male dominated. And that is kind of intimidating and also just kind of a turnoff when it’s like they are just going and grunting and doing their thing, which is fine, I’m OK with that now, I think, but as someone who is more fem presenting and smaller, it was difficult to feel comfortable in that space.”
Not safe	Emotional	“like for like watching yourself and I would say like it has a very bro-y mentality, which. In my personal experience just makes me very uncomfortable because then I feel like I have to show up in a certain way to like to be able to be there. And that just wears out. That wears on me over time.”
Safety	Physical body	“One, obviously, from a safety perspective, like it’s just learning the lifts and getting giving someone a feeling of mastery because that in and of itself can be a good thing. I think, like good safe lifting involves being hyper aware of one’s body.”
Safety	Physical body	“hey [trauma survivors] need to know that their body can be a safe space. And how to really take care of a body, I think so, from movement to, you know, nutrition to water to rest. I think that those things all get mixed up in trauma in different ways. And so that becomes really important to learn how to connect with a body and how to take care of it.”
Safety	Physical space	“like design, but I would imagine, certain. Layout colors, like often gym’s very overly masculine, like there’s metal, like chrome and all sorts of like black and white, exciting colors and the. You know, maybe like changing that a little bit. I know there’s there’s some like more like these gyms that are have experimented with like the type of music, lighting and stuff like that to create different sort of environments within the gym to varying degrees of success”
Safety	Physical space emotional	“Although it wasn’t like a super huge space, things were spaced out enough that I did not really feel like anyone was on top of me and I could generally maintain my sense of personal boundary. And I also appreciated the space felt kind of like clean and professional, and it seemed from the way it was designed that it was almost intended to be open to any kind of personality type and not a specific one.”
Safety	Physical space emotional	“And so maybe having spaces without mirrors where you could maybe there’s areas with mirrors that are like not prominent or you can go and practice your technique and not be viewed by other people like those sorts of maybe like just structured areas where there’s spaces for privacy and what not. There you where you get a little bit of the best of both worlds.”
Safety	Physical space emotional	“And just, you know, knowing where the exits are, you know, being able to visualize the door and things like that and to always kind of had that invitation that if you need to leave, there is a place where you can go and that there’s a place where you can sit and be by yourself to some extent, you know, or to sit and get, you know, to be able to have a space outside of the exercise room where you can regroup a little bit if you need that.”
Safety	Emotional	“I think a lot of athletic language is gendered and I know we do not have new terms for that. But exploring what that kind of means like there’s men’s and women’s bars. So does that mean we have the lighter bar the heavier bar? I do not know. Exploring what that means. I do not think there’s necessarily a certain look, but it’s more in the language we choose to use and or like. It’s like there’s the prescribed workout. Like you do the men’s workout and a women’s workout. And like there’s some guys that are not that big and the women’s workout is just fine for them. So, yeah, just exploring what that looks like and allowing people to feel safe. And it’s better for everyone because if there is a smaller guy, he’s like you. But using a women’s bar, there’s no shame in that. But like we are culturally taught to like feel embarrassed about it”
Safety	Physical space emotional	“And it would be really nice to have that option at a gym, for example, just having a space where it’s just maybe women and not a mixture of men and women. And having just that privacy so that I can move my body the way I need to and not feel like I’m an object or being stared at or this feeling of sexualization.”
Safety	Physical space emotional	“even though I do not identify as a queer person, the fact that kind of friendliness generated a pretty welcoming and accepting environment, even if you did not belong to any of those categories, was something that benefited from and allowed me to use that space as a training ground to build a fair bit of confidence.”
Safety	Emotional coaching	“he’s very intentional about where he puts himself. And I think that because I asked him about it, because I noticed that he was because he used to sit the gym or gym, moved locations, but he used to always sit outside on the railing, which is very strange. But I like ask him about that. And it’s because he was like intentionally putting himself somewhere where people could see him. He’s not ever sneaking up on someone like, you know where he is. And I think that, like, that was helpful, especially at the beginning when I was very anxious when he would come to coach me. I was very [inaudible] with it. I’d never had someone like watch you move. I did not want people to watch me move. Was just really uncomfortable.

### Researcher positionality/reflexivity

Despite the authors’ positive intentions and collective commitment to ensuring findings remain rooted in participant experiences, we acknowledge the potential impact of privileged identities held by authors both individually and collectively. All members of the research team engaged in ongoing discussion and analysis of emerging themes considering team members’ positionalities.

## Findings

### Participants

The study included 46 participants who ranged in age from 23 to 68 years of age; the average age was 36 years old. Table 2 provides an overview of participant demographics collected via survey. All questions were optional, thus N-values differ across categories. A majority of the sample were white women with college degrees or higher. Twenty-six percent of participants identified as transgender, nonbinary, or gender nonconforming, and 35% identified as lesbian, gay, bisexual, queer, or questioning. The participants had lifted weights an average of 9 years (range 2 years to 40 years). Most participants resided in urban centers and hailed from the American Northeast, Mid-Atlantic, Midwest, South, and West. Ninety-three percent of participants had worked with a coach, mostly in the gym setting. However, COVID-19 restrictions forced many to lift weights at home, and 37% of respondents reported lifting weights occasionally or entirely at home. Seventy percent of respondents reported working with a weight lifting coach at least once. Eighty-six percent of the sample reported interpersonal trauma, 36% reported intrapersonal trauma, and 31% reported systemic trauma. Participants were able to select more than one trauma type, thus percentages for this response total to greater than 100%. When the trauma types were combined, 33% of the sample reported experiencing one trauma type, 52% reported experiencing two trauma types, and 15% reported experiencing all three trauma types. Only 3% reported the trauma to be an isolated incident (acute), whereas 65% reported the trauma experiences to be repeated over time (chronic), and 32% of the sample reported both an isolated incident and additional trauma experiences that were repeated over time.

**Table 2:** Participant demographics.

Demographic	Response	Percentage
Gender identity (N = 42)		
	Woman	55%
	Man	19%
	Nonbinary	10%
	Transgender man	13%
	Agender	2%
Race (N = 37)		
	White	65%
	Black	3%
	Hispanic/Latinx	14%
	Asian/Pacific Islander	5%
	Multiracial	13%
Sexual Orientation (N = 39)		
	Straight	51%
	Queer	18%
	Lesbian	5%
	Bisexual	13%
	Questioning	3%
	Asexual	3%
Education (N = 37)		
	Some college	14%
	College degree	51%
	Graduate degree	35%
Employment (N = 42)		
	Full time	65%
	Part time	21%
	Not working	14%
Trauma Type (N = 36)		
	Interpersonal	86%
	Impersonal	36%
	Systemic	31%
	Other	8%
Trauma Frequency (N = 31)		
	Acute	3%
	Chronic	65%
	Both	32%
Weight Lifting Setting (N = 42)		
	Gym	50%
	Home	18%
	Both	32%

In semi-structured, one-on-one interviews, additional qualitative information was obtained. While some participants reported an ability to access psychotherapy, others reported barriers to mental health treatment such as cost and stigma. Some participants reported histories of housing insecurity or houselessness, exposure to neighborhood violence, and poverty. Some participants also described disruptions in their ability to continue weight lifting due to financial constraints. Among those participants who accessed mental health treatment, most reported that having a professional who understood trauma and offered trauma-informed interventions, such as eye movement desensitization and reprocessing (EMDR) therapy, promoted psychological healing. Participants also cited lifestyle interventions, such as having a routine, healthful habits (nutrition, sleep, exercise) and social connection as contributors to healing. Several participants self-reported the use of other coping mechanisms to manage their trauma symptoms including substance use, self harm/self injury, and disordered eating patterns. While some participants came from a youth or collegiate sports background, others reported no prior experience with exercise or sports before weight lifting.

Interviews with trauma-impacted weight lifters also elucidated the multiple facets of a weight lifting program that become particularly consequential in the aftermath of psychological trauma. Several themes emerged across interviews– pertaining to the gym environment, coaching strategies, gym community, and relationship with self and coach– that highlight opportunities for rethinking standard approaches to weight lifting. While many possible organizational frameworks could be offered to convey these findings, we found that the salient analytic categories mapped onto existing evidence-based frameworks for trauma-informed interventions. Through a deductive approach, we found these themes could be organized according to the six principles of trauma-informed care outlined by SAMHSA: safety; trustworthiness, and transparency; peer support; collaboration and mutuality; empowerment, voice, and choice; and cultural, historical, and gender issues. Thus, this research both validates SAMHSA’s model of trauma-informed care and subsequently draws upon extending and rebuilding it into a trauma-informed weight lifting framework to analyze respondents’ narratives.

### Safety

Safety emerged as a salient theme in the discussion of trauma’s effects on the body and mind. Numerous respondents described a lack of a sense of safety in their own body in the aftermath of trauma. The implications of this sense of safety spanned relationships with self, relationships with others, and respondents’ ability to participate in weight lifting.

“They [trauma survivors] need to know that their body can be a safe space. And how to really take care of a body, I think so, from movement to, you know, nutrition to water to rest. I think that those things all get mixed up in trauma in different ways. And so that becomes really important to learn how to connect with a body and how to take care of it.”

Safety as it pertains to weight lifting was discussed in relation to the gym environment, relationships with coaches, and the physical act of weight lifting. Elements of the gym environment shaped respondents’ sense of safety in the space. For instance, respondents reported exacerbations in symptoms of hypervigilance when detecting frequent movement in their visual fields through mirrors in the gym. In addition, the sudden noise of dropping weights startled some respondents and provoked anxiety. For other respondents, loud music projected over gym speakers was unsettling. A lack of private space also increased feelings of vulnerability for some respondents.

“I know for me, I don't like a space where everyone– like a fishbowl– everyone is walking by and looking in. So let's say we have a room that's designated for a class, but then there's windows everywhere and everyone's just walking by and looking in. That is an awful feeling. I don't like that at all. That's horrible. I don't I don't feel safe in that environment.”

Respondents also described social and cultural aspects of the gym environment that made them feel unsafe, including a predominant culture of masculinity or aggression and the exclusion of people of color, women, and LGBTQ+ community members in the gym.

“I struggled with the idea of the concept of weight lifting when I was younger because it was so cis and het and white, I want to say primarily, and like very cis-male dominated. And that is kind of intimidating and also just kind of a turnoff when it's like they're just going and grunting and doing their thing, which is fine, I'm OK with that now, I think, but as someone who is more fem presenting and smaller, it was difficult to feel comfortable in that space.”

For some respondents, the lack of a sense of safety in the gym environment was a barrier to participation in weight lifting. However, respondents also described positive aspects of the gym environment that promoted their sense of safety and facilitated their access to weight lifting. These included positive experiences with the physical space which could make gyms feel more welcoming to people of all identities.

“…blue and white, were probably the predominant colors, not very, I would say it's more calming than on the aggressive side. Not a lot of decor with like, you know, skulls and things like that that you might find at a powerlifting meet. And I also appreciated the space felt kind of like clean and professional, and it seemed from the way it was designed that it was almost intended to be open to any kind of personality type and not a specific one.”

Respondents also noted that the layout of the gym could also promote their sense of safety in the space.

“Although it wasn't like a super huge space, things were spaced out enough that I didn't really feel like anyone was on top of me and I could generally maintain my sense of personal boundary. I've been in a lot of gym settings where I feel kind of in the past because they're very busy and they're packed and both in terms of the way the equipment is distributed and also with the way the equipment is distributed in that way, that people are also distributed in that way. And those settings tend to make me feel really encroached upon in a way that sets off my alarm systems a little bit.”

### Trustworthiness and transparency

The theme of trustworthiness and transparency emerged most prominently in respondents’ discussions of their relationships with coaches. Respondents emphasized the importance of explicit expectations and boundary-setting in establishing trust in the coaching relationship. In addition, collaboration with coaches regarding programming and the responsiveness of coaches to weight lifters’ needs were important contributing factors to trust in the coaching relationship.

“I think they'd have to build trust in the relationship. I think it's really important to feel like your coach sees you as a person with unique needs and abilities and tailors programming.”

Respondents identified the need for programming to be both tailored to each individual client and also responsive to the client’s needs during a particular training session.

“I think that trainers or coaches need to be prepared to adjust the movements on the fly to meet somebody where their nervous system is that day.”

The flexibility of coaches to make adjustments to programming in response to clients’ trauma-related concerns was also an important theme among respondents.

“The other thing that I don't love is people standing over me while I'm laying down, which can be hard. And because if someone is teaching you how to bench press, how are they gonna do that? So I do think I learned how to do incline bench press first, which it's like you're sitting more up and the person is standing more behind you instead of looming over you. And that was really good for me.”

Transparency from coaches regarding the logic of programming also emerged as a crucial component of a trusting professional relationship. A supportive environment in which to try, fail, and succeed promoted weight lifers’ sense of confidence and belonging.

“The very first time that I ever tried [intense interval training], I went to a gym where I had a little bit of exposure to some weight lifting, but not a lot. And the instructor with the prescribed weights said that's too much for you without ever explaining or without seeing what I was capable of. And I left that experience feeling very rejected and more stirring up some of those old even traumas of insecurity and like, oh, I'm not enough. And contrast that to, the second I went to a different place. The second experience where it was like, what? Try it. Let's see. Let's see what you can do. And that it was supported and encouraged. And I couldn't do the initial. It was like, yeah, that's too heavy. I can't lift that. But that it was, well, let's find out what you can do and so much more of just like that encouragement. And that was so healing to my heart to be recognized as like, let's see, let's test it out there. There isn't a negative here.”

Many respondents described the importance of negotiating physical touch as a crucial component of transparency and boundary-setting in the coaching relationship.

“I also think about more specifically how you spot somebody is really important. And spotting is a really important part of weight lifting. And also I've been in so many weight lifting spaces where coaches or helpful standers-by are touching people's bodies without their consent. Or spotting in ways that aren't the only option, but happen to be an option that don't feel safe for that person. So I think about squatting, right, and so, you know, having your hands on someone's waist is by no means the only way to support, you know, spot someone’s squat. And guess what? I don't want it. So some of the interpersonal things attending to what needs someone has in their body. I think a lot about not reproducing abusive dynamics, being really aware of the power dynamics of having whatever coaches or health professionals or whomever might be in that space or querying those, and naming them and making them transparent.”

Respondents also identified the need for multiple accepted avenues for the communication of boundaries, including both verbal and nonverbal communication methods.

“People don't always want to be touched, but they don't always want to stand out as the one that's like waving their hands around saying they don't want to be touched either. So I guess this is more yoga than weight lifting, but I've had like yoga instructors that have been like, *put your palms down if you don't wanna be touched* or something like that.”

### Peer support

Numerous respondents described the importance of community in transforming weight lifting into a therapeutic practice. Broadly, a sense of community in the gym was felt to contribute to healing from trauma.

“I think that is another important component, especially if one is depending on the trauma of one's feeling isolated. If you go to the gym enough, you do start to see the same people and you start, you know, you have your gym friends and those are good.”

Without a sense of community within the gym, some respondents described feeling too unsafe to perform certain lifts.

“Every time you go to the gym, it's a different set of people. I mean, sometimes you'll see people you know or you'll get in a routine. But most of the time it's a different group of people every time. And so– I don't know you. I don't know what you're capable of. You know, all of those fears and you're vulnerable especially if you're on one of those, if you're doing a chest press and you're on your back, your body is just laying there for everyone to see. It's pretty exposing.”

Respondents also described the potential role of gym community members to identify and intervene in instances in which trauma-impacted gym-goers may benefit from support. Frequently, these instances involved experiencing psychological or bodily harm through weight lifting.

“Over-exercising was actually really positively reinforced and encouraged by people around me rather than maybe being recognized or like no one got curious about what that was about.”

“And then while I was training in that sport, I experienced an acute trauma and the subsequent PTSD and my training became very, very compulsive. And I was training, weight lifting and then powerlifting. And I had also taken up karate in there. And I had started strength training many years prior to help take care of some chronic problems. But where I was training, which was like training to fight, I was always in fight, it was pretty harmful to my health. It was celebrated by the fitness community that I was acting like a machine. And no one was like, hey, why don't you rest? So I wound up severely injured and it was a real struggle to get back to training.”

### Collaboration and mutuality

Respondents described the diversity of traumatic experiences among weight lifters and noted that some approaches to weight lifting that may address the needs of one participant may not address the needs of another. As such, respondents called for a collaborative approach to designing weight lifting spaces that solicits and responds to feedback from stakeholders. One respondent reflected on how weight lifting gyms could attend to the diverse needs of clients.

“How does it feel? Does it have natural light yet a sense of privacy? That's really nice. It's ideal if you can have your ideal space. Right. It's like privacy. And for my adjustable light, you know. No one agrees on sounds, things like if you're doing Olympic weightlifting, those hours maybe you want to express other clients who are very sensitive to sudden noises, like from the hours of this Olympic training facility like this, because I have seen clients who have worked with me and barbell clubs who have come for like a session. And even if it's just one person lifting every time that bar hits the floor, they start. There's only so much you can address. But just to even be aware that this could be a thing and to keep an open mind, the music that is triggering for some may not be triggering for others. We don't know what triggers are going to be for people. I guess it's all part of the training as well for the staff, but also things like a community agreement. I think also when you design the space, all stakeholders should be represented. So that involves people who represent a potential community as well as the staff.”

To respondents, effective collaboration with clients required coach knowledge of trauma-related topics.

“If a coach is able or better able to modify or recognize signs that someone might be struggling with something without them having to tell them, that I think would be really beneficial or has been beneficial.”

Some respondents who were also weight lifting coaches described a similar practice for promoting collaboration.

“I try to do this in my own consults with clients. Is touch base on like if ever you feel uncomfortable about something like please let me know, like I want to be able to accommodate you and like, make you feel comfortable and safe because this is your time and like your practice. So you should be in charge of it. And so, like touching base with clients ahead of time on that, those things never really delving into like what are your traumas, but more like if anything triggers you, if anything is harmful in some way, let me know so I can help correct that or help you manage that in the space. And I think establishing that from the beginning is like the most important, because that usually, I think is what people want to hear when they walk in like you are safe here, like what you need, you just let us know and we will try to give you what you need.”

### Empowerment, voice, and choice

Respondents emphasized the importance of choice and autonomy in weight lifting. The lack of choice in programming often negatively impacted respondents’ healing trajectories.

“Some of the moments that weight lifting was harmful in my life was, I really think about the training protocol that I was on before that tournament. I had no autonomy. I was really isolated. It was not a program that was designed around my body, the environment that I had to weight lift in was like not super supportive or savvy. And so in that moment, if I had had access to my own wisdom and anything I wanted, that's not the kind of physical activity and movement that would have been best for my healing, not because of weight lifting, but because of the container.”

Respondents described the need for choice and tending to individual needs in group classes.

“Choice, which is, you know, that's part of the trauma informed is choice. And that's huge. And so I have had classes where they're looking at me in a crowd of people because I'm choosing to do something different with my body and I regardless, I don't listen to them and I do what's good for my body, but that's really intimidating.”

“Well, the first time I started with a trainer, she was like I was like, this is what I want. And she was like, OK. But the YMCA says, I have to measure your BMI and take all these physical measurements and your body fat percentage. And this has to be part of the routine. So, I mean, if you think about that, like I'm coming in, you know, as someone who has had, you know, a lot of experiences of not being in control of my body and I'm wanting to have this positive experience. But first, that, you know, this is now this hoop that I have to jump through. And the first time I went along with it, even though it made me feel really bad, it made me feel really triggered because I just like was OK. This is the thing that I have to go through to do this, you know?”

### Cultural, historical and gender issues

Respondents frequently described the impact that messaging and representation in gym environments had on their sense of safety and belonging in the gym. Respondents with multiple intersecting identities described instances of exclusion and re-traumatization in gym environments.

“I think about the toxic masculinity and the incredible fat phobia and weight stigma that exists in weight lifting spaces as something that was incredibly hard on my body and really impeded healing or caused distress in many moments.”

“It has always been like I'm like fighting physical embodiments of toxic masculinity in order to make it like to get to a bar. And I think I certainly have some, like, emotional wounds and some emotional tenderness as a result of navigating those environments.”

In response to such experiences, respondents called for inclusivity surrounding body shape and size, gender identity, sexual orientation, and race and ethnicity in gym environments. Inclusivity involved access to facilities designed with historically marginalized groups in mind.

“But then I think about some of the things along with like, you know, it would be really, really trauma informed in the weight lifting space that I access is gender inclusive changing rooms. Like gender inclusive locker rooms, gender inclusive bathrooms, right, because part of the really specific trauma and shit that I experience in weight lifting spaces that I'm not into is binary ism and like transphobia and like experiencing specific harassment because I'm a non binary person trying to access sport related spaces that are so binary.”

“It would be really nice to have that option at a gym, for example, just having a space where it's just maybe women and not a mixture of men and women. And having just that privacy so that I can move my body the way I need to and not feel like I'm an object or being stared at or this feeling of sexualization.”

Representation and inclusivity included spanned symbolic messaging to staff demographics.

“Having images of folks of various body shapes and sizes, of various physical ability or capacity and color, of course, and gender that already makes people feel comfortable when you first walk in. Having trainers that are not all just the same looking as well, again, just from a visual standpoint. Having that, or like signage or like expressing that in the way that one markets, I guess, and like being an advocate for certain movements and being active in encouraging people to lift without necessarily pushing them to change their body in a way that's for everybody else and not for them.”

“…recruiting coaches who actually reflect the diversity of humans who experience trauma. Both in terms of finding coaches who have lived experience and also that, you know, I would love to have at any time worked with someone who is not a cis het white man.”

Respondents also noted that responsiveness to cultural, historical, and gender issues promoted a welcoming environment more broadly.

“Even though I don't identify as a queer person, the fact that that kind of friendliness generated a pretty welcoming and accepting environment, even if you didn't belong to any of those categories, was something that I benefited from and allowed me to use that space as a training ground to build a fair bit of confidence.”

## Discussion

Research supports the centrality of the body and movement in trauma healing, yet research exploring the necessity and helpfulness of taking a trauma-informed approach to various movement and exercise practices is limited. Core to all trauma-informed practice is inclusion of the voices and needs of trauma survivors, which has served as inspiration for this study as it aims to elevate and center the experiences of weight lifters who have experienced trauma to further inform how to take a trauma-informed approach in weight lifting spaces and coaching relationships.

In the current study, findings indicate the need for a trauma-informed, anti-oppressive and inclusive approach in the gym environment when working with participants who have experienced psychological trauma and with marginalized and minoritized identities. Respondents shared their experiences feeling unwelcome in gym spaces because of institutional practices and norms that reinforce broader cultural norms that are discriminatory towards certain identities and body types, particularly within fitness and wellness culture. Respondents also expressed a need for greater physical and emotional safety which some identified could be attended to through changes in both the physical and social environment of the weight lifting gym. These changes ranged from a reduction in mirrors to the availability of gender-neutral facilities to the inclusion of body-positive messaging. Although to our knowledge, no studies have evaluated the perspectives of trauma-impacted populations regarding psychological safety in gym environments, these findings support the results of qualitative research that has indicated that historically marginalized populations, such as women, experience significant psychosocial barriers to accessing the gym (Clark, 2018).

Additionally, respondents endorsed the need for safety in the context of relationships with coaches. Respondents called for a greater understanding of psychological trauma among coaches accompanied by flexible and responsive approaches to programming. In addition, respondents highlighted the importance of clear expectation and boundary setting in the coaching relationship and the value of attentiveness to communication across a variety of verbal and nonverbal modalities. These findings support recent proposals for trauma-informed coaching techniques in youth sports (Bergholz et al., 2016) and underscore the need for similar considerations among trauma-impacted adult populations engaged in weight lifting.

The results of this study reveal that peer support is a critical component of trauma healing and may play a unique role in the formation of trauma-informed weight lifting spaces. Respondents expressed feelings of isolation in the aftermath of trauma and noted the role that gym communities could play in promoting a sense of safety and inclusion in the gym. Prior research has described how sharing similar experiences, receiving acceptance from peers, and responding to opportunities to help others, help to counter feelings of isolation and promote self-worth and self-esteem among group members (MacKenzie, 1998). Research has also shown that social connection among members of a group is a powerful predictor of empowerment (Davis et al., 2015). However, respondents underscored the fact that gym communities that are unfamiliar with the manifestations of trauma could inadvertently reinforce distressing behaviors and coping mechanisms among trauma-impacted weight lifters. These findings indicate the need for trauma-informed initiatives to include not only coaches, but to engage whole gym communities.

Collaboration and participant voice and choice emerged as important features of the coaching relationship. Respondents described psychologically harmful experiences with protocolized training programs and expressed the need for greater collaboration between coach and client in determining and pursuing goals. Examples include the choice to opt-out of weigh-ins and body mass index (BMI) measurements and greater collaboration with coaches to offer a wider array of modifications during group classes. These findings support the results of prior research, which suggest that encouraging clients to choose a level of movement based on how they feel in a specific situation helps foster mind-body connection (Emerson et al., 2009). This research also suggests that techniques intended to emphasize participant voice and autonomy in sports coaching with youth impacted by trauma may also be relevant when working with adult weight lifters (Bergholz et al., 2016).

Finally, cultural, historical, and gender issues were evident in respondents’ calls for greater inclusivity surrounding body shape and size, gender identity, sexual orientation, race, and ethnicity in gym environments. Respondents cited examples of feeling unsafe or unwelcome in gym environments because they did not conform to the normative expectations most pervasive in the gym or fitness industry at large. Respondents urged for more inclusive spaces and messaging in gyms to shift the culture of weight lifting towards greater inclusion of people of diverse identities. These findings align with the results of prior research which has demonstrated the ways in which cultural, historical, and gender issues result in the exclusion of marginalized populations from the gym and other arenas for physical exercise (Denison et al., 2021; Herrick et al., 2022). It must be noted that although the study population was well-representative of some populations historically excluded in gym spaces, such as women and members of the LGBTQ+ community, it was also disproportionately white. As several respondents of color reported during interviews, the disproportionately white gym environment had at times served as a barrier to their participation in weight lifting. These findings underscore the need for continued anti-racist work within weight lifting and trauma healing spaces.

Taken together, the findings from the current study support the development of specific guidelines and recommendations for trauma informed coaching with lifters impacted by trauma. The following guidelines are based on the principles of trauma-informed care and contribute to a nascent but growing research agenda regarding the development of trauma-informed weight lifting programming (Bergholz et al., 2016; Nowakowski-Sims et al., 2023).

Research participants’ narratives indicate that a trauma-informed approach to weight lifting must be premised upon a recognition of trauma’s disproportionate impact on individuals and groups who experience marginalization and oppression, including people of color, women and those assigned female at birth, members of the LGBTQIA+ community, people of size, people with disabilities, and those living in poverty and experiencing socioeconomic disparities. Research participants reported both experiences of exclusion and harm in gym environments as well as experiences of healing and growth. Thus, a trauma-informed approach to weight lifting must both address the harms present in gym environments and promote the healing potential of weight lifting. To do so, we suggest that trauma-informed weight lifting adapt and evolve traditional anti-oppressive and trauma-informed frameworks and principles to account for important context-specific considerations that arise in the gym setting.

We propose a two-step framework for trauma-informed coaching that begins with harm-reduction initiatives. Motivated by research participants’ calls for greater inclusivity within gym environments as well as advocacy from within the interdisciplinary field of trauma-informed research, such a framework should build upon a foundational knowledge of trauma and incorporate anti-oppression (Havig and Byers, 2019) and anti-racist (Powell et al., 2022) frameworks and values to increase inclusion and reduce experiences of harm and retraumatization within the gym environment and coaching relationships. The second step in this proposed framework includes trauma-informed coaching techniques as well as considerations for individual weight lifting practitioners to support trauma healing and resilience. Beginning with harm-reduction principles, we propose the following features of a training and practice model:

*Foundational Knowledge of Trauma:* Beginning with an understanding of the definitions, language and neurobiology of trauma along with contextual and historical research explaining trauma’s rootedness in systems and institutions as well as the intergeneration, historical, cultural and identity based nature of many traumas and adversities. Additionally, we support a deepening awareness of individual and collective positionality within the frameworks of privilege, access, and power. The diverse and varied manifestations of trauma across domains (e.g., intrapersonal, interpersonal, physiologically/physically, behaviorally, emotionally, psychologically, etc.) are not only understood, but embraced in an effort to depathologize trauma and normalize its manifestations as reasonable and necessary coping and adaptation strategies.

*Inclusivity*: With an understanding of the negative impact of mainstream fitness culture on bodies and identities, practitioners must respect the stories, experiences, abilities, bodies and needs that are not their own. Practitioners should be supported in efforts to find and bolster pathways to limit barriers or access and to actively combat and change toxic fitness culture narratives and practices (i.e., diet culture, ableism, “no days off” mentality, etc.).

*Practitioner Self Awareness:* It is paramount that practitioners of all kinds, including coaches and personal trainers, do their own inner examination with their relationship to power and privilege in order to not replicate trauma paradigms that we have all been indoctrinated in. Active and ongoing work must be done to increase awareness around implicit and explicit biases and to engage in reflective practice.

In taking a stance to first minimize opportunities for harm in an effort to reduce retraumatization and combat experiences of adversity, discrimination, oppression and harm, work is then conducted to offer transformative and healing experiences and practices:

*Responsivity in Relationship:* The impacts of trauma are well documented. At the center are relationships and how power moves or can be manipulated in the context of relationships between individuals, groups, and within systems. Cultivating relational dynamics where no one is coerced or manipulated is paramount to trauma-informed care. Embodying responsivity in relationship requires the practitioner to actively observe, seek information from those they work with, and respond according to needs and requests within their scope of practice and in attuned ways.

*Stance of Curiosity:* Responsive practice also requires a willingness to ask questions and be curious. Trauma-informed practice in action looks like wondering about how individuals and groups communicate in explicit and implicit ways about their histories, experiences and needs. Practitioners should foster a practice of wondering about how a person or people have adapted to, survived, and coped with their experiences versus taking a hierarchical and power over approach. Additionally, trauma-informed practitioners must remain curious about themselves and actively seek to become more self-aware.

*Interoceptive Awareness:* Trauma can have negative impacts on one’s ability to connect with one’s sensations, body and internal experiences. As a result, supporting the development of interoceptive awareness (the ability to notice and understand internal signals and sensations as helpful and necessary information) further supports autonomy, nervous system regulation, and self-attunement. Practitioners should encourage the use of weight lifting and resistance training as a mechanism for increasing interoceptive awareness.

*Agency, Autonomy and Choice:* Trauma robs individuals of choice, control and agency. Healing from trauma requires that practitioners center agency, autonomy and choice at every step of practice to ensure an individual and group’s ability to identify their own needs and develop self-trust through the practice of weight lifting and movement. Practitioners should promote the cultivation of weight lifting spaces and communities where individual and collective needs dictate how movement is approached, language used, and the physical environment is constructed.

*Healing Relationships and Community*: In addition to supporting connection to self, a trauma-informed approach to weight lifting encourages connection with others in community as healing relationships further address the impacts of trauma and foster greater resilience. Healing relationships and communities also use intentional processes to address harm through accountability and restorative/transformative practices.

## Conclusion

The firsthand accounts of trauma-impacted weight lifters described above are instructive for the evidence-based design of trauma-informed weight lifting spaces and programming. By responding to the express needs of trauma-impacted groups, it may be possible to increase the accessibility of weight lifting as a therapeutic resource for those seeking healing, particularly for those groups historically excluded from weight lifting spaces. Formal recommendations for trauma-informed programming also create opportunities for certifying bodies to include trauma-informed instruction in their curricula.

Grounded in the experiences of weight lifters impacted by trauma, the recommendations offered as part of this study represent the first published, evidence-based recommendations for trauma-informed weight lifting. However, this study is not without its limitations. This study was conducted with a small sample size with limited representation of certain demographics, including men, people of color, and people with disabilities. Thus, the results of this study may not be generalizable to these groups. Future research should explore the lived experiences and specific needs of these populations with larger sample sizes. As this research is theoretical in nature, subsequent studies should explore the feasibility and efficacy of these recommendations under controlled experimental conditions.

## Data availability statement

The raw data supporting the conclusions of this article will be made available by the authors, without undue reservation.

## Ethics statement

The studies involving human participants were reviewed and approved by Barry University Institutional Review Board. The patients/participants provided their written informed consent to participate in this study.

## Author contributions

EN-S, DV, MR, and SW contributed to the conception and design of the study, conducted the interviews, transcribed the interviews, and performed the data analysis. EN-S organized data collection and analysis. DV wrote the first draft of the manuscript. MR and EN-S wrote sections of the manuscript. All authors contributed to the article and approved the submitted version.

## Conflict of interest

The authors declare that the research was conducted in the absence of any commercial or financial relationships that could be construed as a potential conflict of interest.

## Publisher’s note

All claims expressed in this article are solely those of the authors and do not necessarily represent those of their affiliated organizations, or those of the publisher, the editors and the reviewers. Any product that may be evaluated in this article, or claim that may be made by its manufacturer, is not guaranteed or endorsed by the publisher.

## Appendix

### Interview questions

- Tell me a little about yourself and your experience with weightlifting? How long/what types/individual or group
- What sorts of things do you think individuals who have experienced trauma (like you) might need for their health? What about their mental health? What about just lifestyle and well-being?
- What has been most helpful in your healing?
- What are your beliefs about exercise and weight lifting benefitting overall health? What are your beliefs about mind-body connection?
- Ask only if they note a positive connection: How has weight lifting impacted your healing from past trauma?
- What is your perception of any changes (positive, negative, or both/ neither) through participation in weight lifting? What’s different? How are you different? In what ways is your life different because of weight lifting?
- Do you see any value in having a gym where trauma-related needs and problems are taken into account?
- What specific elements (programming, coach-participant relationship, setting, etc) have been more/less beneficial? What kinds of things should a coach/trainer/gym do when working with lifters with a trauma history?
- Is there anything we should have talked about, but did not?
